# ELISA is superior to bacterial culture and agglutination test in the diagnosis of brucellosis in an endemic area in China

**DOI:** 10.1186/s12879-019-4729-1

**Published:** 2020-01-06

**Authors:** Nannan Xu, Wei Wang, Fengzhe Chen, Wen Li, Gang Wang

**Affiliations:** 1 Department of Infectious Disease, QiLu Hospital, Shandong University, Jinan, 250012 Shandong China; 2 Department of Hematopathology, The University of Texas MD Anderson Cancer Center, Houston, TX 77030 USA

**Keywords:** Brucellosis, ELISA

## Abstract

**Background:**

Brucellosis is endemic in many areas in China. The current diagnosis of Brucellosis predominantly relies on the traditional bacterial culture and serum agglutination test. In this study, we aimed to explore the value of ELISA in the diagnosis of Brucellosis in Chinese population.

**Methods:**

We recruited 235 patients with a diagnosis of Brucellosis at different clinical stages: 117 in acute, 78 in subacute, and 40 in chronic. We also recruited 248 control patients who presented with similar clinical symptoms but with a different diagnosis other than Brucellosis. In addition, 90 healthy volunteers were also recruited. Bacterial culture, agglutination test and ELISA assay were performed to detect Brucella spp.

**Results:**

Among 235 patients with Brucellosis, 51 (21.7%) was positive for bacterial culture, 150 (63.8%) were positive by agglutination test, and 232 (98.7%) were positive by ELISA (IgG and/or IgM). When we stratified the patients based on the disease stages (acute, subacute and chronic), ELISA was the most sensitive method and showed a highest positive rate in all stages. By Receiver Operating Characteristic Curve analysis of ELISA results, we found that measurement of IgG level was superior to measurement of IgM level (AUC, 0.993 versus 0.877). Since the measurement of IgG itself missed rare cases in acute phase, we recommended measuring IgG and IgM simultaneously by ELISA for the diagnosis of Brucellosis. In term of the specificity of ELISA in the diagnosis of Brucellosis, our study showed that only 1.6% (4/248) non-Brucellosis patients were positive by ELISA; all positive cases were IgM only and none showed positive IgG. Similar results were found in healthy volunteers. In summary, our study concluded that ELISA is the most sensitive and specific method to detect Brucellosis in Chinese population.

**Conclusions:**

ELISA assay is sensitive, fast, and convenient to detect Brucellosis. It shows the high sensitivity and specifity and should be used as a routine lab test when Brucellosis is suspected in clinical practice.

## Background

Brucellosis, also called Mediterranean fever, is zoonotic infectious disease caused by Brucella spp. It infects humans as well as animals such as sheep, cattle, goats, pigs, and dogs. The bacteria that cause human infection include three main types: B. melitensis, *B. abortus*, and B. suis with B. melitensis being the most common [1]. In recent years some new species were also identified [2–5]. Brucellosis is transmitted from animals to humans in several ways. The most common route of transmission occurs when humans consume raw milk or cheese made from infected animals. The disease can also be transmitted to humans via inhalation of the organism or by direct contact with secretions or feces from infected animals. Patients infected with Brucella often present with fever, sweating, arthralgia, hepatosplenomegaly and lymphadenopathy.

In China, the occurrence rate of Brucellosis has increased significantly since 1990s and it gradually becomes one of the most prevalent infectious diseases. In 2016, 47,139 cases were reported with the incidence rate of 3.44/100,000. Northern China is the main endemic area [6]. In addition to China, many other areas such as the Mediterranean Basin, Mexico, Eastern Europe, Africa and the Middle East are also at high risk for Brucellosis. Given the high prevalence of Brucellosis, a rapid and reliable test for the diagnosis is necessary.

Brucellosis is treatable but early diagnosis followed by timely medical intervention is the key. Currently the diagnosis of Brucellosis in China relies heavily on blood culture and serum agglutination test [7–9].. Patients in acute phase often show a higher positivity rate than patients in subacute and chronic phases [10, 11], but due to the overall low sensitivity, negative blood culture can not rule out Brucellosis. Serum agglutination test is used in many hospitals but it also has its own problems; since most areas in China are endemic, there is a low level of antibody titer in normal population, making it difficult to set up a threshold to balance the sensitivity and specificity, which causes some false positive as well as false negative results. ELISA kit for the detection of Brucellosis is commercially available. It is fast, convenient and can detect both IgG and IgM to bacterial surface antigens, and thus can be potentially used as an effective tool to detect Brucellosis [12]. To date, tthere is no systematic study in China to compare ELISA to traditional methods such as blood culture and agglutination test. In this study, we aimed to evaluate the value of ELISA in the diagnosis of Brucellosis and compared it to the assays currently used in clinical practice.

## Methods

### Study cohort

We recruited two groups of patients admitted to our hospital between May 2016 and August 2018. The patients were from northeast part of China. Group 1 included 235 patients who were diagnosed with Brucellosis. The diagnosis of Brucellosis was based on the proper clinical context, including history (occupationally exposed or consumption of raw dairy/meat product or living endemic areas), clinical presentation (fever, sweating, arthralgia, hepatosplenomegaly) and laboratory studies as well as at least one of the following results being positive: bacterial culture, agglutination test or ELISA test. Group 2 included 248 patients who were admitted with similar clinical presentations but later confirmed to have diseases other than Brucellosis (systemic lupus erythematosus, viral and bacterial infection). Besides these two patient groups, we also recruited 90 healthy volunteers as controls. All clinical information including age, sex, clinical presentation, laboratory studies, treatment history and contact history was collected.

This study is approved by the ethics committee of Qilu Hospital. All patients and volunteers signed the consent forms.

### Bacteria culture

Patients’ blood were collected and transferred to blood culture bottles (Bactec plus/F; Becton Dickinson, Franklin Lakes, NJ, USA), which were incubated in the Bactec system (Becton Dickinson Diagnostic Instrument Systems, NJ) until a positive result was obtained or for a maximum of 10 days. The isolates were identified based on Gram-negative coccobacilli, urease and oxidase positivity and positive agglutination with specific antiserum.

### Serum agglutination test

The standard tube agglutination antigen was purchased from Center for Disease Control and Prevention, China. Patient serum was serially diluted from 1:10 to 1:1280 using phenol saline. Brucella antigen was added and the mixture was incubated at 37 °C for 24 h. All tubes were compared with control tubes (positive and negative controls) to examine agglutination. Titers ≥1:100 with a minimum of 50% agglutination were considered positive.

### ELISA test

ELISA kit was purchased from IBL Intermational GmbH, Germany. ELISA assay was performed following the manufacture’s instructions and the cutoff value for positive antibody test is ≥12u/ml. Briefly, for IgG detection, patients’ serum was diluted at 1:10, and 100ul diluted serum was added to each well for incubation for 1 h. After washing, enzyme-conjugated reagent was added for 30 min. After another round of washing, the substrate for enzyme was added for 20 min. Stop buffer was added and OD value was measured at 450 nm. Standard curve was established using the OD values from controls. The value of tested samples was calculated based on the standard curve. For the detection of IgM antibody, the procedure is similar with an extra step of pre-absorption before the procedure.

### Statistical analysis

GraphPad Prism 7.0(GraphPad, La Jolla, CA, USA) was used for data analysis including calculating the sensitivity, specificity, false negative and false positive values. For evaluation of diagnostic value of IgM and IgG, receiver operating characteristic curve (ROC curve) and area under the curve (AUC) was established. Paired x^2^ test was used for comparison between agglutination test and ELISA test. *P* < 0.05 was considered statistically significant.

## Results

### Clinical characteristics

The clinical characteristics of Brucellosis patients and control patients were listed in Table 1. Patients with the diagnosis of Brucellosis were further subclassified as culture-positive group and culture-negative group, and their clinical characteristics were listed in Table 2. As shown in Table 2, there was no statistical difference in terms of clinical and laboratory findings among these two groups. Among Brucellosis patients at the time of their initial admission, 117 (50%) was in acute stage (< 8 weeks), 78 (33%) was in subacute stage (8–24 weeks) and 40 (17%) was in chronic stage (> 24 weeks). The duration of fever before initial admission ranged from 6 days to 2 years. Among Brucellosis patients, 138 (58.7%) were occupationally exposed, including farmers, veterinarians, and dairy-industry professionals et al., and the remaining patients either lived in endemic areas or had a history of consumption of raw dairy or meat products.

**Table 1:** Demographic and clinical characteristics of Brucellosis patients and Non-brucellosis control patients

Characteristics	Brucellosis patients (*n* = 235)	Non-brucellosis controls(*n* = 248)	P
Mean age (mean ± SD), years	50.97 ± 17.98	51.95 ± 17.54	0.598
Male	131 (55.74%)	138 (55.65%)	0.982
Fever	218 (92.77%)	209 (84.27%)	0.004
Joint pain	162 (68.94%)	130 (52.42%)	< 0.001
Hepatosplenomegaly	124 (52.77%)	143 (57.66%)	0.279
WBC (mean ± SD), × 10^9^/L	5.66 ± 0.90	6.73 ± 3.08	0.007
CRP (M, IQR), mg/L	19 (4.51, 33)	24 (14, 48)	< 0.001
PCT (M, IQR), ng/L	0.25 (0.16, 0.34)	0.46 (0.23, 1.02)	< 0.001

**Note:** Data presented as numbers (%) unless otherwise indicated. WBC:white blood cells; CRP:C-reactive protein; PCT:Procalcitonin

**Table 2:** Demographic and clinical characteristics of culture-positive and culture-negative Brucellosis patients

Characteristics	Culture-positive Brucellosis (*n* = 51)	Culture-negative Brucellosis (*n* = 184)	*P* value
Mean age (mean ± SD), years	48.39 ± 19.96	51.68 ± 17.38	0.248
Male	28 (54.90%)	103 (56.0%)	0.891
Fever	45 (88.23%)	173 (94.02%)	0.269
Joint pain	32 (62.75%)	130 (70.65%)	0.280
Hepatosplenomegaly	25 (49.02%)	99 (53.80%)	0.545
WBC (mean ± SD), ×10^9^/L	5.82 ± 0.90	5.62 ± 0.90	0.160
CRP (M, IQR), mg/L	23 (8.01,42.0)	18 (3.85, 32)	0.037
PCT (M, IQR), ng/L	0.234 (0.128, 0.32)	0.26 (0.166,0.345)	0.116
Focal involvement	24 (47.06%)	78 (42.39%)	0.552
Osteoarticular involvement	13 (25.49%)	50 (27.17%)	0.810
Genitourinary involvement	5 (9.8%)	15 (8.15%)	0.928
Neurological involvement	2 (3.92%)	3 (1.63%)	0.297
Pulmonary involvement	4 (7.84%)	10 (5.43%)	0.758

### Laboratory findings

The results of blood culture, agglutination test and ELISA assay are listed in Table 3. Among 235 Brucellosis patients, blood culture was positive in 51 (21.7%) patients and agglutination test was positive in 150 (63.8%) patients. In comparison, ELISA test demonstrated a high positive rate of 98.7% (232/235) (IgG and/or IgM positive). Statistical analysis using McNemar x^2^ test showed ELISA was superior to blood culture and agglutination test for detection of Brucellosis (*P* < 0.01). When analyzing IgG and IgM separately, the overall positivity rate for IgM by ELISA was 60.9%, and the antibody level ranged from 1.07 to 83.7 U/ml (median 16.81 U/ml with quartile Q1 and Q3: 7.96 U/ml and 28.85 U/ml respectively). In contrast, the overall positivity rate for IgG was higher at 96.2%, and the antibody level ranged from 2.09 to 700 U/ml (median 42.83 U/ml with quartile Q1 and Q3: 22.02 U/ml and 78.55 U/ml respectively). In ELISA assay, only 3 (1.3%) patients showed negative results for both IgG and IgM (<12u/ml). Among these 3 patients, 1 showed positive blood culture and negative agglutination test; this patient had a relatively short disease duration with 6 days of fever, and two weeks later, a repeated ELISA showed positive IgM and IgG results. The remaining 2 patients with negative ELISA result were also negative by blood culture but positive by agglutination test, and both patients had several rounds of empirical antibiotic therapy prior to the admission to our hospital.

**Table 3:** Results of Culture, STA and ELISA performed on 235 brucellosis patients

Cases	STA: *N* (%)		ELISA: IgM and/or IgG *N* (%)	
	Positive	Negative	Positive	Negative
Total (*N = 235*)	150 (63.83%)	85 (36.17%)	232 (98.72%)	3 (1.28%)
Culture positive (*N* = 51, 21.70%)	41 (80.39%)	10 (19.61%)	50 (98.04%)	1 (1.96%)
Culture negative (*N* = 184, 78.3%)	109 (59.24%)	75 (40.76%)	182 (98.91%)	2 (1.09%)

**Note:** STA: standard tube agglutination

In 248 control patients, no patients showed positive culture for Brucella spp. Agglutination test was positive in 14 (6.45%) patients. ELISA was positive in 4 (1.61%) patients and all were IgM positive only, no patients in this group showed positive IgG by ELISA. The IgM antibody level by ELISA in these control patients ranged from 0.5 to 49.58 U/ml (median 2.75 U/ml with quartile Q1 and Q3: 1.49 U/ml and 4.86 U/ml respectively) and IgG antibody level ranged from 0.5 to 11.8 U/ml (median 2.835 U/ml with quartile Q1 and Q3: 2.033 U/ml and 4.158 U/ml respectively).

In order to assess the background antibody titer in normal population, we recruited 90 adult healthy individuals for ELISA assay. One (1.1%) showed elevated IgM at 30.8 U/ml and the antibody level ranged from 0.5 to 30.8 U/ml (median 2.34 U/ml with quartile Q1 and Q3: 1.528 U/ml and 4.31 U/ml respectively) and. None showed elevated IgG and the antibody level ranged from 1.03 to 10.93 U/ml (median 2.27 U/ml with quartile Q1 and Q3: 1.718 U/ml and 3.153 U/ml respectively).

We then combined and compared all results from Brucellosis patients, control patients and normal healthy controls and calculated the sensitivity and specificity. As shown in Table 4, ELISA showed the higher sensitivity (0.987) and specificity (0.984) when compared to agglutination test (sensitivity 0.638, specificity, 0.935) (*p* < 0.001 and *P* = 0.012 respectively).

**Table 4:** Sensitivity, Specificity, True Positive and True Negative values of various tests

	Sensitivity (95%CI)	Specificity(95% CI)	PPA (95%CI)	NPA (95%CI)
Culture	0.217 (0.167,0.276)	1.000 (0.981,1.000)	1.000 (0.913,1.000)	0.574 (0.526,0.621)
STA	0.638 (0.573,0.0.699)	0.935 (0.895,0.961)	0.903 (0.845,0.942)	0.732 (0.679,0.779)
IgM by ELISA	0.609 (0.543,0.671)	0.984 (0.956,0.995)	0.972 (0.927,0.991)	0.726 (0.675,0.773)
IgG by ELISA	0.961 (0.926,0.981)	1.000 (0.981,1.000)	1.000 (0.979,1.000)	0.964 (0.932,0.983)
IgM + IgG by ELISA	0.987 (0.960,0.997)	0.984 (0.956,0.995)	0.983 (0.954,0.994)	0.987 (0.962,0.997)

**Note:** STA: standard tube agglutination. PPA: positive percent agreement. NPA:negative percent agreement

### Laboratory findings at different stages of brucellosis

We next focused on Brucellosis group only and analyzed the laboratory results based on disease stages (acute, 117 cases; subacute, 78 cases; and chronic stages, 40 cases). The results of blood culture, agglutination test and ELISA test were summarized in Table 5. Positive blood culture was identified in acute stage (36.8%, 43/117) and subacute stage (10.3%, 8/78) only, and no patients in chronic stage were tested positive by blood culture. In agglutination test, the positivity rate showed a similar trend with the highest positive rate in acute phase (75.2%) followed by 57.7% in subacute phase and 42.5% in chronic phase. In contrast, ELISA showed high positive rates in all stages of disease: 98.3% in acute, 100% in subacute and 97.5% in chronic stage. Statistical analysis using McNemar x^2^ showed ELISA is superior to detect Brucellosis in all stages of diseases when compared to blood culture and agglutination test (*p* < 0.001 in both cases).

**Table 5:** The results of blood culture, STA and ELISA in different stages of Brucellosis (number and the percentage of positive cases by each assay)

Group	Acute (< 8 weeks) *N* = 117	Subacute (8–24 weeks) *N* = 78	Chronic (>24 weeks) *N* = 40
Culture: *N*(% positivity)	43 (36.75%)	8 (10.26%)	0 (0.00%)
STA: *N*(% positivity)	88 (75.21%)	45 (57.69%)	17 (42.50%)
IgM by ELISA: *N*(% positivity)	93 (79.49%)	43 (55.13%)	7 (17.50%)
IgG by ELISA: *N*(% positivity)	110 (94.02%)	78 (100%)	38 (95.00%)
IgM and/or IgG by ELISA: *N*(% positivity)	115 (98.30%)	78 (100%)	39 (97.50%)

**Note:** STA: standard tube agglutination

### The different value of IgM and IgG measured by ELISA assay in the diagnosis of brucellosis

**Fig. 1:**
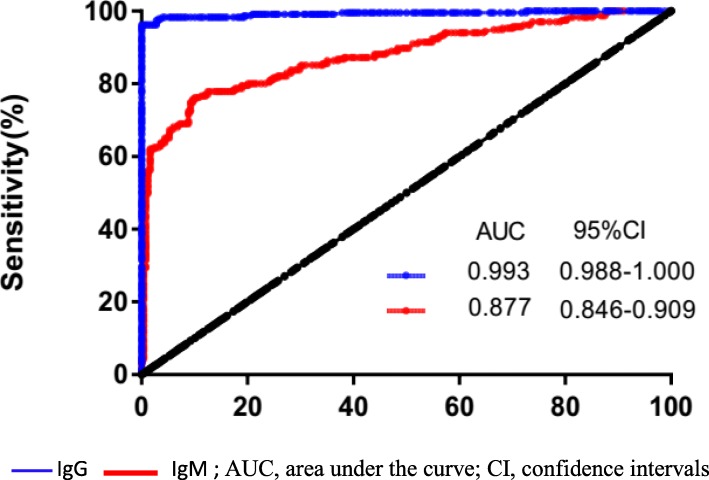
Receiver Operating characteristic analysis was performed for IgG and IgM to determine threshold values for discriminating between Brucellosis and Non-brucellosis

As mentioned earlier, we measured IgM and IgG simultaneously using ELISA. As shown in Table 5, IgM and IgG showed different positive rates. The positive rate of IgM decreased as the disease persisted and prolonged: 79.5% in acute phage, 55.1% in subacute phase, and 17.5% in chronic phase. In contrast, the positive rate of IgG remained at a high level in all phases of the disease: 94% in acute phase, 100% in subacute phase and 95% in chronic phase. We evaluated the diagnostic value of IgM and IgG using ROC curve with the calculation of AUC. As shown in Fig. 1, the AUC value for IgG is 0.993 (95% CI, 0.988–1.000), higher than the AUC value of IgM (0.877 with 95% CI, 0.846–0.909). The sensitivity and specificity of IgM and IgG were calculated using GraphPad. As shown in Table 4, the sensitivity was 0.609 for IgM and 0.961 for IgG, and the specificity was 0.984 for IgM and 1.00 for IgG. In summary, the overall diagnostic value of IgG is superior to IgM. Of note, IgG failed to detect the disease in 5 acute patients with positive IgM, thus the simultaneous measurement of IgM and IgG will yield the best diagnosis value.

### Clinical follow-up

Patients were treated with anti-Brucellosis agents after the diagnosis of Brucellosis. We followed up the patients 2, 4, and 6 weeks after the treatment. Among 235 Brucellosis patients, 10 lost follow-up including 2 from culture-positive group and 8 from culture-negative group. Their clinical symptoms, complete blood counts, liver and kidney function, inflammatory parameters as well as the compliance of medications were collected. We summarized their clinical response and listed in Additional file 1: **Table S1**. As illustrated in the table, most patients responded the treatment very well with rare patients who showed treatment failure or relapsed disease.

## Discussion

In China, the incidence rate of Brucellosis has increased with a relatively rapid pace since 1990s. During the last 10 years, the occurrence rate has steadily increased approximately 7.8% annually [13]. Based on the evaluation from WHO, the actual number of Brucellosis patients is much higher, approximately 10–25 times of reported cases [14]. This big discrepancy between the reported rate and the actual incidence rate is largely due to the misdiagnosis and underdiagnosis, especially in endemic areas. For Brucellosis, the gold standard diagnostic assay is bacterial culture. However, the culture tends to be negative in subacute and chronic stages. Among 235 consecutive patients recruited in this study, more than half of patients were already beyond acute stage (> 8 weeks). As a result, the positive rate of blood culture is only 21.7%, similar to the results published previously [15, 16].

The main serology study currently used in China is agglutination test. Similar to bacterial culture, the positivity of agglutination test decreases as the disease prolongs [17, 18]. In our study, the positive rate was below 50% in chronic stage (Table 5). Even in acute stage with positive bacterial culture, patients can have false negative results [19, 20], (19.6% in our study). Another potential problem for agglutination test is the presence of cross reactivity with other bacteria, such as Yersinia enterocolitica, Salmonella urbana group N, Vibrio cholera, and Francisella tularensis, causing false positivity [7]. Finally, the official diagnosis criterion for Brucellosis in China is > 1:100 with obvious agglutination (> 50%), lower than the criteria proposed by WHO (> 1;160). Given many areas in China are endemic, there is a background positivity in normal population, which may lead to false positive results (6.45% in this study).

In this study, we demonstrated that ELISA has higher sensitivity and specificity to detect Brucellosis than agglutination test, consistent with a few previous studies [21–23]. In culture positive cases, the positive rate for ELISA is 98% and the positive rate for agglutination test is 80.4%. As the disease progresses, the positive rate for culture and agglutinin test decreases substantially while ELISA still keeps its high positive rate. This is particularly important because many patients (50% in current study) in China present at subacute and chronic stages when initially admitted and at these stages blood culture as well as aggulutin showed low positivity.

Between IgG and IgM by ELISA, IgG showed a better diagnostic utility with a higher sensitivity and specificity. The elevated IgG is not seen in patients with other diseases and normal healthy people. In contrast, IgM elevation can be rarely seen in other diseases; in this study, 2 cases with autoimmune disease showed elevated IgM. In 90 healthy controls, we also found 1 case with elevated IgM. Although IgG is superior to IgM, simultaneous measurement of IgG and IgM is recommended as IgG can be rarely negative in acute stage; in our study, 5 cases of Brucellosis initially presented with isolated IgM elevation with no elevated IgG level; all were in acute phase and after 1-month follow-up, IgG changed to positive. Thus, for patients in acute phase with negative IgG, we recommend repeating the test after 2–4 weeks. Of note, two cases in our study were negative for both IgM and IgG by ELISA during the initial presentation as well as later follow-ups; one had 3 weeks of disease duration and the other had 8 months. Both patients had a history of antibiotics treatment before admission. Previous studies have indicated that anti-bacterial therapy can decrease the antibody titer in ELISA assay [24, 25]. Consistently, our follow-up study demonstrated that the antibody titer decreased significantly 2 months after therapy in patients with positive ELISA result at initial diagnosis (data not shown). Thus the prior antibiotics treatment is likely the cause of false negative result in ELISA assay. Although ELISA shows high sensitivity and specificity for the diagnosis of Brucellosis, we should be aware that it is an antibody-based test, thus patients’ immune status and background antibody titer in normal population in endemic areas can affect the assay and potentially cause some false negative or false positive results. We recommended that interpretation of ELISA results should be incorporated with clinical and laboratory findings.

## Conclusion

In summary, using a large cohort composed of 235 Brucellosis patients, 248 control patients and 90 healthy individuals, we demonstrated that ELISA has the highest sensitivity and specificity to detect Brucellosis at all stages. It is superior to blood culture as well as agglutination test. Given its rapid turnaround time and relatively simple and standardized protocol, we strongly recommended using ELISA test in daily clinical practice when Brucellosis is in the differential diagnosis. This is particularly valuable in China and many other endemic areas as many patients in these areas have subacute or chronic stages as the initial presentation.

## Supplementary information

**Additional file 1: Table S1.** Clinical follow up of patients with a diagnosis of Brucellosis

## Data Availability

The datasets used and/or analysed during the current study are available from the corresponding author on reasonable request.

## Abbreviations

AUC: Area under curve
ELISA: Enzyme-linked immunosorbent assay
ROC: Receiver operating characteristic
WHO: World health organization
