# Supplementary material for: ELISA is superior to bacterial culture and agglutination test in the diagnosis of brucellosis in an endemic area in China
Source: BMC Infect Dis. 2020 Jan 6;20:11. doi: 10.1186/s12879-019-4729-1 (PMC6945397; doi:10.1186/s12879-019-4729-1)
Supplement: Supplementary file 1 — Additional file 1: Table S1. Clinical follow up of patients with a diagnosis of Brucellosis [file 12879_2019_4729_MOESM1_ESM.docx]

Supplementary Table 1: Clinical follow up of patients with a diagnosis of Brucellosis

|  | Culture-positive Brucellosis (n=49) | | Culture-negative Brucellosis(n=176) | |
| --- | --- | --- | --- | --- |
|  | Without focal involvement  (n=25) | With focal involvement^a^  (n=24) | Without focal involvement  (n=100) | With focal involvement  (n=76) |
| Clinical response^b^ | 23 (92.00%) | 22 (91.67%) | 94 (94.0%) | 72 (94.74%) |
| Treatment failure^c^ | 0 (0.00%) | 1 (3.70%) | 1 (1.0%) | 0 (0.00%) |
| Relapse^d^ | 2 (8.00%) | 1 (4.17%) | 5 (5.0%) | 4 (5.26%) |

Focal involvement^a^: Involvement of specific organs with the presence of organ-related symptoms

Clinical response^b^: Improvement of clinical symptom and laboratory abnormalities

Treatment failure^c^: No improvement at 4 weeks after the treatment

Relapse^d^:patients showed response, but relapsed after the treatment was ceased.
